# Correction: Hoshi et al. High Correlation among Brain-Derived Major Protein Levels in Cerebrospinal Fluid: Implication for Amyloid-Beta and Tau Protein Changes in Alzheimer’s Disease. *Metabolites* 2022, *12*, 355

**DOI:** 10.3390/metabo13060685

**Published:** 2023-05-25

**Authors:** Kyoka Hoshi, Mayumi Kanno, Mitsunari Abe, Takenobu Murakami, Yoshikazu Ugawa, Aya Goto, Takashi Honda, Takashi Saito, Takaomi C. Saido, Yoshiki Yamaguchi, Masakazu Miyajima, Katsutoshi Furukawa, Hiroyuki Arai, Yasuhiro Hashimoto

**Affiliations:** 1 Department of Biochemistry, Fukushima Medical University, Fukushima 960-1295, Japan; khoshi@fmu.ac.jp; 2 Department of Forensic Medicine, Fukushima Medical University, Fukushima 960-1295, Japan; kannoma@fmu.ac.jp (M.K.); ponchan@fmu.ac.jp (T.H.); 3 Department of Neurology, Fukushima Medical University, Fukushima 960-1295, Japan; mitzabe@gmail.com (M.A.); maaboubou@gmail.com (T.M.); ugawa@fmu.ac.jp (Y.U.); 4 Center for Integrated Science and Humanities, Fukushima Medical University, Fukushima 960-1295, Japan; agoto@fmu.ac.jp; 5 Laboratory of Proteolytic Neuroscience, RIKEN Center for Brain Science, Saitama 351-0198, Japan; saito-t@med.nagoya-cu.ac.jp (T.S.); takaomi.saido@riken.jp (T.C.S.); 6 Structural Glyocobiology Team, RIKEN Global Research Cluster, Saitama 351-0198, Japan; yyoshiki@tohoku-mpu.ac.jp; 7 Department of Neurosurgery, Juntendo University, Tokyo 113-8421, Japan; mmasaka@juntendo.ac.jp; 8 Institute of Development, Aging and Cancer, Tohoku University, Miyagi 980-8575, Japan; katsfuru@hotmail.com (K.F.); hiroyuki.arai.b5@tohoku.ac.jp (H.A.)

## Error in Table

In the original publication [1], there was a mistake regarding Tables 1 and 2 as published. We examined the correlation between biomarkers, which should have been analyzed via Spearman’s correlation instead of Pearson’s correlation method. The paper reported that cerebrospinal fluid glycoproteins, such as Man-Tf, GlcNAc-Tf, and PGDS were highly correlated to each other in three stages of Alzheimer’s disease (AD): cognitively normal (CN), mild cognitive impairment (MCI), and AD. The high correlation of the three markers is shown in a new Table 1, which is essentially similar to the previous Table 1. The paper also reported that three markers highly correlated with AD core markers, namely p-tau, tau, and Abeta40 at the CN stage, and that Man-Tf and GlcNAc-Tf were correlated with p-tau and tau at the MCI stage. Three markers were correlated with p-tau at the AD stage. The results are shown in a new Table 2, which is essentially similar to the previous Table 2. Thus, our conclusions are supported by the data in the new tables. The corrected Table 1 and Table 2 appear below.

## Text Correction

There was an error in the original publication. We examined correlations among biomarkers, which should have been analyzed by Spearman’s correlation method instead of Pearson’s correlation.

A correction has been made to Section 2, “Results”; Section 2.4, “Correlations between Major CSF Proteins in CSF of CN, MCI, and AD”; and Section 2.5, “Correlations between Major CSF Proteins and AD Biomarkers”.

### 2.4. Correlations between Major CSF Proteins in CSF of CN, MCI, and AD

Correlations of the CSF proteins were also analyzed for the CN, MCI, and AD clinical groups. In CN, Man-Tf levels correlated with GlcNAc-Tf (*rs* = 0.86) and L-PGDS (*rs* = 0.47) levels (Table 1), while GlcNAc-Tf also correlated with L-PGDS (*rs* = 0.57). In contrast, Man-Tf levels did not correlate with Sia-Tf or TTR levels. High correlations were thus found among Man-Tf, GlcNAc-Tf, and L-PGDS in the CN group. In MCI, Man-Tf levels correlated well with GlcNAc-Tf (*rs* = 0.73) and L-PGDS (*rs* = 0.66) levels, while GlcNAc-Tf correlated well with L-PGDS (*rs* = 0.69). Man-Tf levels were negatively, but weakly, correlated with TTR (*rs* = −0.36) levels but not with Sia-Tf. Again, high correlations were observed among brain-derived proteins in the MCI group. In AD, Man-Tf levels correlated well with GlcNAc-Tf (*rs* = 0.79) and L-PGDS (*rs* = 0.62) levels but not with Sia-Tf or TTR. On the other hand, GlcNAc-Tf levels correlated well with L-PGDS (*rs* = 0.61) levels. These results suggest that the brain-derived proteins showed significant correlations with each other across all clinical groups.

### 2.5. Correlations between Major CSF Proteins and AD Biomarkers

Our previous study demonstrated that Man-Tf and p-tau were co-expressed in hippocampal neurons in the AD brain and that CSF Man-Tf levels correlated well with p-tau levels in MCI and AD. Other CSF proteins, such as Man-Tf, GlcNAc-Tf, L-PGDS, TTR, and Sia-Tf, were therefore examined for their correlation with AD markers (Table 2). In CN subjects, the CSF proteins showed significant correlations with AD markers as follows: Man-Tf vs. p-tau (*rs* = 0.56), tau (*rs* = 0.56), Aβ40 (*rs* = 0.73), and Aβ42 (*rs* = 0.52); GlcNAc-Tf vs. p-tau (*rs* = 0.76), tau (*rs* = 0.79), Aβ40 (*rs* = 0.76), and Aβ42 (*rs* = 0.49); L-PGDS vs. p-tau (*rs* = 0.84), tau (*rs* = 0.79), Aβ40 (*rs* = 0.79), and Aβ42 (*rs* = 0.52). The Aβ42/Aβ40 ratio showed negative correlations with Man-Tf (*rs* = −0.15), GlcNAc-Tf (*rs* = −0.34), and L-PGDS (*rs* = −0.44); however, none of these were statistically significant. In contrast, neither Sia-Tf nor TTR levels correlated with any AD markers. Taken together, the brain-derived CSF proteins correlated significantly with p-tau, tau, Aβ40, and Aβ42 levels in the CN group but the blood-derived proteins did not. In MCI, CSF protein levels also correlated with AD markers as follows: Man-Tf vs. p-tau (*rs* = 0.71), tau (*rs* = 0.53), Aβ40 (*rs* = 0.45); GlcNAc-Tf vs. p-tau (*rs* = 0.65), tau (*rs* = 0.54), and Aβ40 (*rs* = 0.43); L-PGDS vs. p-tau (*rs* = 0.49) and tau (*rs* = 0.41). The Aβ42/Aβ40 ratio showed negative correlations with Man-Tf (*rs* = −0.24), GlcNAc-Tf (*rs* = −0.45), and L-PGDS (*rs* = −0.23) but none were statistically significant. Overall, in the MCI group, the brain-derived proteins correlated moderately with AD markers. In AD, p-tau showed a moderate, though significant, correlation with the CSF proteins (*rs* = 0.51~0.55) but not with other AD markers. TTR negatively correlated with p-tau (*rs* = −0.42) and tau (*rs* = −0.72), while Sia-Tf correlated with tau (*rs* = −0.41). These results suggest that AD markers tend to correlate with the brain-derived CSF proteins in the AD and MCI groups; however, with the exception of p-tau, their correlation declined concomitantly with AD progression.

The authors state that the scientific conclusions are unaffected. This correction was approved by the Academic Editor. The original publication has also been updated.

## Figures and Tables

**Table 1:** Correlation coefficients among CSF major proteins in CN, MCI and AD.

**CN**		**Man-Tf**	**GlcNAc-Tf**	**L-PGDS**	**TTR**	**Sia-Tf**
**Man-Tf**	*rs*	1.00	0.86 ***	0.47 *	−0.02	−0.02
**GlcNAc-Tf**	*rs*	0.86 ***	1.00	0.57 **	0.05	0.18
**L-PGDS**	*rs*	0.47 *	0.57 **	1.00	0.12	0.63 **
**TTR**	*rs*	−0.02	0.05	0.12	1.00	0.51 *
**Sia-Tf**	*rs*	−0.02	0.18	0.63 **	0.51 *	1.00
**MCI**		**Man-Tf**	**GlcNAc-Tf**	**L-PGDS**	**TTR**	**Sia-Tf**
**Man-Tf**	*rs*	1.00	0.73 ***	0.66 ***	−0.36 *	0.05
**GlcNAc-Tf**	*rs*	0.73 ***	1.00	0.69 ***	−0.29	0.20
**L-PGDS**	*rs*	0.66 ***	0.69 ***	1.00	−0.11	0.34 *
**TTR**	*rs*	−0.36 *	−0.29	−0.11	1.00	0.19
**Sia-Tf**	*rs*	0.05	0.20	0.34 *	0.19	1.00
**AD**		**Man-Tf**	**GlcNAc-Tf**	**L-PGDS**	**TTR**	**Sia-Tf**
**Man-Tf**	*rs*	1.00	0.79 ***	0.62 ***	−0.02	−0.02
**GlcNAc-Tf**	*rs*	0.79 ***	1.00	0.61 ***	0.06	0.03
**L-PGDS**	*rs*	0.62 ***	0.61 ***	1.00	−0.15	0.38 **
**TTR**	*rs*	−0.02	0.06	−0.15	1.00	0.40 **
**Sia-Tf**	*rs*	−0.02	0.03	0.38 **	0.40 **	1.00

Spearman’s rank correlation coefficients (*rs*) among brain-derived glycoproteins are highlighted in yellow. Man-Tf data of MCI and AD are cited from our previous paper [15]. Probability levels: * *p* < 0.05, ** *p* < 0.01, *** *p* < 0.001.

**Table 2:** Correlation coefficients among CSF major proteins and AD biomarkers in CN, MCI and AD.

**CN**		**p-tau**	**tau**	**Aβ40**	**Aβ42**	**Aβ42/Aβ40**
**Man-Tf**	*rs*	0.56 *	0.56 *	0.73 **	0.52 *	−0.15
**GlcNAc-Tf**	*rs*	0.76 ***	0.79 **	0.76 ***	0.49 *	−0.34
**L-PGDS**	*rs*	0.84 ***	0.79 ***	0.79 ***	0.52 *	−0.44
**TTR**	*rs*	0.09	0.14	0.01	0.08	0.02
**Sia-Tf**	*rs*	0.36	0.33	0.27	0.10	−0.32
**MCI**		**p-tau**	**tau**	**Aβ40**	**Aβ42**	**Aβ42/Aβ40**
**Man-Tf**	*rs*	0.71 ***	0.53 **	0.45 **	0.41	−0.24
**GlcNAc-Tf**	*rs*	0.65 ***	0.54 **	0.43 *	0.40	−0.45
**L-PGDS**	*rs*	0.49 **	0.41 *	0.29	0.40	−0.23
**TTR**	*rs*	−0.19	−0.24	−0.27	−0.40	0.12
**Sia-Tf**	*rs*	0.24	0.03	0.31	0.46	−0.15
**AD**		**p-tau**	**tau**	**Aβ40**	**Aβ42**	**Aβ42/Aβ40**
**Man-Tf**	*rs*	0.55 **	0.18	−0.02	0.00	0.00
**GlcNAc-Tf**	*rs*	0.51 **	0.25	−0.08	0.32	0.21
**L-PGDS**	*rs*	0.53 **	0.15	0.05	0.39	0.32
**TTR**	*rs*	−0.42 *	−0.72 ***	−0.09	−0.07	−0.32
**Sia-Tf**	*rs*	−0.17	−0.41 *	0.25	0.54	0.25

Spearman’s rank correlation coefficients (*rs*) values higher than 0.50 are highlighted in yellow between brain-derived major glycoproteins and AD biomarkers. Man-Tf data of MCI and AD are cited from our previous paper [15]. Probability levels: * *p* < 0.05, ** *p* < 0.01, *** *p* < 0.001.

## References

[1] Hoshi K., Kanno M., Abe M., Murakami T., Ugawa Y., Goto A., Honda T., Saito T., Saido T.C., Yamaguchi Y. (2022). High Correlation among Brain-Derived Major Protein Levels in Cerebrospinal Fluid: Implication for Amyloid-Beta and Tau Protein Changes in Alzheimer’s Disease. Metabolites.

